# Relaxation and exercise in lymphoma survivors (REIL study): a randomised clinical trial protocol

**DOI:** 10.1186/s13102-019-0127-7

**Published:** 2019-08-16

**Authors:** Suchita Hathiramani, Ruth Pettengell, Hannah Moir, Ahmed Younis

**Affiliations:** 1 Kingston University and St. George’s University of London, London, UK; 2 St. George’s Healthcare NHS Trust, London, UK; 3 Kingston University, Kingston Upon Thames, London, UK; 4 Faculty of Health, Social Care & Education, Cranmer Terrace, London, SW17 0RE UK

**Keywords:** Lymphoma survivors, Exercise, Relaxation, Mindfulness, Rehabilitation, Cancer survivors, Self-management, Quality of life

## Abstract

**Background:**

Lymphoma survivors commonly report ongoing complaints including fatigue, pain, depression and decreased quality of life (QoL) following treatment. Although evidence suggests that both relaxation and exercise can significantly improve such symptoms, there is no consensus on which intervention is more effective. This paper presents the REIL (Relaxation and Exercise In Lymphoma) Study protocol. The REIL study aims to compare the effect of two home-based interventions – relaxation and exercise – on QoL in lymphoma survivors.

**Methods:**

Eligible participants (*n* = 36) will be randomised to a relaxation or exercise programme to perform at least three times per week. The primary outcome measure is QoL, assessed by the European Organisation for Research and Treatment of Cancer QoL Questionnaire Core 30 (EORTC QLQ-C30). Secondary outcome measures include body composition, cardiovascular status, pulmonary function, grip strength, functional exercise capacity (six minute walk test), well-being assessed by the FACT-Lym questionnaire, and psychological status assessed by the Hospital Anxiety and Depression Scale. Total duration of the study will be twelve weeks and outcome measures will be assessed at baseline, six weeks and at the end of the study.

**Discussion:**

It is anticipated that results from this preliminary study will begin to highlight effective pathways to improve QoL following chemotherapy for this population. This will better inform healthcare professionals to optimise QoL of lymphoma patients, and enable a smooth transition from being a cancer patient to survivor.

**Trial registration:**

The REIL study has been registered on a publicly accessible database, ClinicalTrials.gov, Registration Number: NCT02272751, October 2014.

## Background

Significant improvements have been made in the survival rates in cancer patients due to earlier detection and advances in treatment. Although initial research in the field of cancer survivorship focused on tumours such as breast and prostate, dramatic advances in haematological malignancies have also led to increased survival [1], but at a cost of increasing complications. Like survivors of other cancers, lymphoma survivors are at an increased risk of morbidity and adverse effects due to disease and treatment exposures. Commonly reported long-term and late effects of treatment in lymphoma survivors include both physical and psychosocial symptoms such as fatigue, pain, muscle weakness, neuropathies, depression, anxiety and decreased self-esteem; as well as decreased function and quality of life (QoL) [1–3]. Hence there has been a call for further research to address the ongoing needs of lymphoma survivors [1].

When medical treatment is completed successfully and the patient is in clinical remission, the close support from medical staff is usually reduced and the patient is expected to return to ‘normal life’. This transition however does not always occur smoothly and studies have highlighted that post-treatment survivors at this transition phase often feel unprepared and uninformed [1, 3]. Survivors at this phase are sometimes in need of further support and advice on how to progress activity, manage ongoing symptoms and return to pre-morbid status and often do not receive this [2, 3]. There are currently no recommended care pathways for lymphoma patients in remission following treatment, despite the fact that this transition period shortly after treatment has been highlighted to be particularly difficult [4].

This lack of care pathways has been highlighted by the government in the United Kingdom (UK). The National Cancer Survivorship Initiative (NCSI) was launched in 2008 and calls for research into the development of pathways for cancer survivors [5], recommending holistic assessment and personalised care-planning, emphasis on the use of patient-reported outcome measures and a shift towards self-management [6].

Self-management in cancer survivorship has been defined as ‘awareness and active participation by the person in their recovery, recuperation and rehabilitation, to minimise the consequences of treatment, and promote survival, health and well-being” [7]. Increasing numbers of survivors together with limited healthcare resources has led to a focus on self-management as an important approach for cancer survivors. Furthermore, self-management can empower cancer patients, increase their confidence to manage problems associated with disease and treatment, and enhance QoL [8].

Literature indicates that interventions such as relaxation and exercise both have a positive impact on QoL of cancer survivors [9–12]. It has been demonstrated that exercise interventions have a positive impact on physical and psychological factors related to QoL such as peak oxygen consumption, physical functioning, fatigue, self-esteem and social functioning; as well as overall QoL of cancer patients and survivors [9, 10]. Studies on relaxation interventions including mindfulness-based stress reduction and progressive muscular relaxation have also demonstrated significant positive impact on physical and psychological symptoms such as fatigue, pain, anxiety and depression, as well as on QoL in cancer survivors [10–12]. Hence both relaxation and exercise interventions are recommended by organisations providing expert advice and support to cancer patients and survivors such as Lymphoma Action [13] and Macmillan Cancer Support [14].

However in the majority of trials studying the effects of relaxation or exercise on cancer survivors, the benefits have only been relative to a control group; and to rule out potential placebo effects future studies need to specify more rigorous comparison conditions, for instance a control intervention with similar elements that may influence outcome such as attention from study personnel or time spent on the procedure [15, 16]. Hence there has been a call for further studies to move beyond wait-list control groups and to compare with active control or other empirically supported interventions [15, 16], for instance relaxation to exercise. As a result, there have been studies and study protocols looking at comparison of two interventions in cancer survivors, but the majority of these have focused on breast cancer [17, 18] and authors have called for future research to focus on other survivor groups, including haematologic cancer sites [1, 19].

The REIL study aims to address some of these issues highlighted by studying a sample of lymphoma survivors post-chemotherapy, supporting patients during the transition phase, comparison of two interventions, use of patient-reported outcome measures and emphasis on self-management. Results from this preliminary study would provide an indication of efficacy of interventions, and with the findings from the REIL study we aim to build towards the development of evidence-based practice guidelines for lymphoma survivors.

This paper presents the REIL study protocol using SPIRIT 2013 guidelines [20].

### Aims

The primary aim of the REIL Study is to compare the effect of two interventions – relaxation and exercise – on QoL in a sample of lymphoma patients in remission post-chemotherapy. The null hypothesis is that there is no difference in QoL between the relaxation and exercise groups post-intervention.

Secondary aims are to investigate the effects of the two interventions on body composition, cardiovascular status, pulmonary function, muscle strength, functional exercise capacity, well-being and psychological status; and explore perceptions about participation in the post-treatment intervention programme.

## Methods

### Study design

The REIL study is a prospective, randomised, clinical intervention trial. Participants will be randomised to exercise or relaxation intervention. Participants will be assessed at baseline prior to commencing the intervention programme, at six weeks, and at the end of the twelve week intervention.

### Ethical approval

The REIL study has received ethical approval from Camden and Islington National Research Ethics Service (13/LO/1327), who are the responsible for approval of final protocol as well as any modifications or amendments. Local site approval from St. George’s Hospital Joint Research and Enterprise Office (JREO) has also been obtained (13.0108). The JREO is an independent office providing external review and monitoring of any research undertaken at St. George’s NHS Trust in order to maintain clinical research governance guidelines and standards.

The study has been registered on a publicly accessible database, ClinicalTrials.gov, NCT02272751.

### Recruitment

Participants will be recruited from a single specialist clinical setting – the Haematology-Oncology Out-Patient (HOOP) Clinic at St George’s Hospital, London. Assessment for eligibility, recruitment, medical screening and obtaining of informed consent will be carried out by the patients’ medical consultant (RP). In order to encourage participant enrolment, potential participants will be introduced to the principal investigator (SH) who will explain about the study, answer questions and provide with the written participant information sheet to take away and read before making a decision.

### Eligibility criteria

Inclusion criteria include patients with histologically confirmed lymphoma in remission post–chemotherapy, chemotherapy treatment completed within the last six weeks, age 18 years or older, able to give informed consent, good performance status (assessed by the Eastern Cooperative Oncology Group {ECOG} status 0–2) [21] and medically able to carry out an exercise training programme. Patients with active disease, unstable angina or unexplained electrocardiogram, poor performance status (ECOG status 3 or more), pregnancy, difficulty breathing at rest, persistent cough, fever or illness, or any cognitive impairment limiting ability to give informed consent or complete QoL questionnaires will be excluded. Written informed consent will be obtained from all individual participants included in the study.

### Sample size

Sample size was calculated to determine clinically relevant effects on the primary outcome measure, the European Organisation for Research and Treatment of Cancer – QoL core questionnaire (EORTC QLQ-C30, version 3.0). A minimally important difference of 5 to 10 points is generally accepted as clinically meaningful [22], and calculations were based on comparison of means between two groups. Assuming a two-sided significance level level (α) of 0.05, power of 80% (ß = 0.20) and standard deviation from EORTC website reference values [23], it was determined that a sample of minimum 46 participants will be required to detect a significant change in the EORTC summary score, 23 in each intervention group.

### Randomisation

A random allocation list will be prepared by the department statistician, generated using GraphPad randomisation software (GraphPad Prism version 6.04 for Windows, GraphPad software, CA). On entry into the study each participant will be assigned an anonymous ID number and each number will be allocated an intervention (exercise or relaxation) on the list. As all assessment sessions will be carried out by the single principal investigator (SH), it will not be possible to blind the investigator to intervention. Also due to the nature of the intervention participants cannot be blinded to group allocation.

**Fig. 1:**
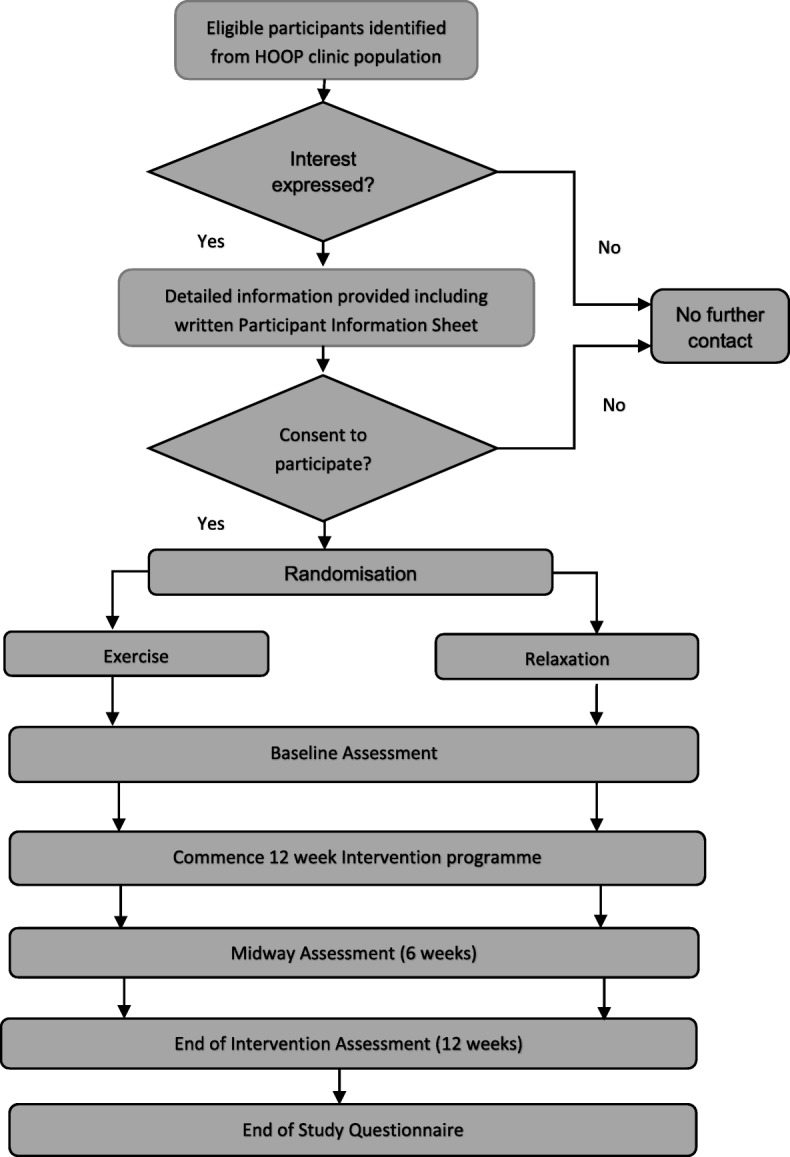
Flow of participants in the REIL Study. CONSORT flow diagram demonstrating participant flow through each phase of the randomised controlled trial (enrolment, intervention allocation, follow-up and data analysis)

Flow of participants in the study is shown in Fig. 1.

### Interventions

Interventions for this study are reported using the template for intervention description and replication (TIDieR) checklist and guide [24].

For both groups, advice, instruction, demonstration and practice will be carried out at baseline. This will be delivered by the principal investigator SH, an experienced physiotherapist with a special interest and experience in oncology. Participants will be able to contact SH by telephone in between sessions if additional support is required. All participants will be advised on potential adverse events and what to report including pain, fatigue, muscle soreness, lymphoedema, nausea, dyspnoea, dizziness, tachycardia and cramp. A logbook will be provided to all participants to encourage adherence and document self-reported participation levels, intervention type carried out, frequency, intensity, duration and any adverse events.

#### Exercise intervention

While there are no guidelines for the delivery of exercise in cancer survivors, it is recommended to follow the guidelines for general UK population [25]. Hence participants randomised to the exercise intervention in this study will be advised to carry out the programme for at least 50 min three times per week.

The exercise programme includes elements of aerobic, upper and lower limb resistance training, core stability and stretches and is designed to be performed independently at home. For the aerobic component, participants will be advised to walk indoors or outdoors for a minimum of 30 min and to aim for moderate intensity as recommended by the Borg Rate of Perceived Exertion (RPE) scale [26]. Where unable to maintain moderate intensity due to fatigue, weakness or other complaints, participants will be advised to aim for moderate intensity aerobic exercise for any duration of time able, and to gradually increase over the next few weeks aiming to build up to 150 min over one week. They will be taught resistance exercises for the major upper and lower limb muscles using body weight or TheraBand™ isometric resistance bands to carry out following aerobic activity. The resistance training follows American College of Sports Medicine (ACSM) guidelines [27], and participants will be advised to carry out three sets of each exercise for 8–12 repetitions. They will also be taught stretches for upper and lower limb muscles, and core abdominal exercises in supine. Participants will be also be provided with written instruction, leaflet with photographs and advice sheet. Similar programmes incorporating aerobic, resistance and flexibility training three times a week over a twelve week period have been used with good results in improving QoL in cancer survivors [9].

#### Relaxation intervention

The relaxation intervention consists of a bed- or chair-based program, developed based on the literature on mindfulness-based interventions in cancer survivors [10–12]. An audio CD was produced to guide participants through relaxation techniques incorporating mindfulness meditation, breathing exercises, guided visualisation and progressive muscle relaxation. Here also participants will be advised to carry out the programme for 50 min three times a week. Participants will also be provided with written information on relaxation and tips on how to incorporate into daily life. Participants in this group will not be advised to perform any exercise outside of their normal habits, nor asked to avoid activity.

Participants will be followed-up at six weeks, midway through the programme. At this stage all assessments will be repeated, any change noted and they will be advised on how to modify the programme (relaxation or exercise) as appropriate. Once participants have completed twelve weeks of their intervention, they will be provided with resources of the other intervention group, for their information or to carry out as desired, in accordance with the Declaration of Helsinki.

### Outcome measures

All outcome measures will be assessed at baseline prior to commencing intervention, midway (six weeks) and on completion of intervention programme (twelve weeks). Outcome measures, instruments and guidelines used and time assessed are summarised in Table 1. At baseline, patient demographics including gender, age, social history and medical history will be recorded.

**Table 1:** Primary and Secondary Outcome Measures

Outcome measured	Instrument	Guidelines	When assessed		
			Baseline	Midway 6 weeks	End of intervention 12 weeks
Primary outcome					
Quality of Life	EORTC QLQ-C30	Self-reported Questionnaire	☑	☑	☑
Secondary outcomes					
Body Composition	Height, Weight, Bioimpedance analysis – BMI, Body Fat % - TANITA BC-418 Body Composition Analyzer	Same investigator and equipment, time of day and clothing kept similar where possible	☑	☑	☑
Cardiovascular status – Resting BP, HR, SpO2	Omron M10-IT electronic BP monitor, Solaris Finger Pulse Oximeter S10A		☑	☑	☑
Pulmonary Function – FEV1, FVC, FER, PEF	Hand-held Micro 1 Medical Microspirometer	British Thoracic Society, 2013	☑	☑	☑
Muscle Strength – Grip Strength	Jamar® Hydraulic Hand Dynamometer	American College of Sports Medicine, 2014	☑	☑	☑
Functional Exercise Capacity	Six Minute Walk Test	American Thoracic Society, 2002	☑	☑	☑
Physical Well-being, Social/Family Well-being, Emotional Well-being, Functional Well-being	FACT-Lym	Self-reported Questionnaire	☑	☑	☑
Anxiety and Depression	HADS		☑	☑	☑
Feelings and Perceptions about participating in programme	End of Study Questionnaire	Open-ended Questionnaire mailed to participants’ home			☑

#### Quality of life

The primary outcome measure is QoL, assessed by the EORTC QLQ-C30. This self-reported questionnaire has been demonstrated to be a valid and reliable tool, takes approximately eleven minutes to complete and most subjects require no assistance [28]. This has been used extensively in cancer survivorship research, and also specifically with lymphoma patients [29]. The EORTC QLQ-C30 consists of thirty items. Each question is answered on a four-point scale and scores are derived according to the EORTC scoring manual [30].

#### Body composition

Due to the illness itself and treatment and side-effects (including steroid use, loss of appetite, nausea and vomiting), lymphoma patients may experience a fluctuation in weight and body fat percentage during the duration of their treatment. Therefore body composition will be assessed using a bioelectrical impedance analyser (Tanita BC-418) to monitor whether it will stabilise to within the desired range. Participants’ standing stature will be measured using a stadiometer (SECA, Germany). Height measured will be inserted into the body composition analyser to obtain weight, body mass index (BMI), and body fat percentage. Participants will be measured without footwear and in light clothing using the same equipment each time.

#### Cardiovascular and pulmonary status

Resting Blood Pressure (BP) and Heart Rate (HR) will be assessed using an electronic BP monitor (Omron M10-IT, Japan) and finger pulse oximeter (Solaris S10A, China) in sitting. In addition, measures of pulmonary capacity and function will be assessed using a hand-held microspirometer (Micro 1 Medical Microspirometer, England) following British Thoracic Society guidelines [31]. Forced expiratory volume in one second (FEV1), forced vital capacity (FVC), forced expiratory ratio (FER) and expiratory peak flow (PEF) will be recorded.

#### Muscle strength

Grip strength is a good indication of global muscle strength and assessment of grip is common method that is used to assess general strength characteristics [32]. Here, isometric grip strength will be measured in kilograms using an adjustable hand-held dynamometer (Jamar***®*** Hydraulic Hand Dynamometer, USA) with the elbow flexed at 90 degrees and the forearm and wrist in neutral position following standardised American College of Sports Medicine (ACSM) guidelines [32].

#### Functional exercise capacity

Due to the variation in the population being studied in terms of age, co-morbidity and physical ability, a submaximal test was considered appropriate for this study. The Six-Minute Walk Test (6MWT) is considered a good indicator of functional ability and exercise capacity and is a common outcome measure to evaluate progress in rehabilitation programmes [33]. This test was chosen as it can be undertaken in a clinical setting with ease, takes a short duration of time to complete and appeared appropriate for this sample of patients following chemotherapy who are generally deconditioned and fatigue quickly. The 6MWT has been validated and used extensively in a variety of patient populations, and has been recommended for use in cancer patients also [34].

The 6MWT will be carried out following guidelines recommended by the American Thoracic Society (ATS) [33]. The test will be carried out in a 30 m marked distance, and the total distance covered in 6 min will be recorded, as well as post-test Heart Rate and oxygen saturation, RPE and Rate of Dyspnoea [26]. The test will cease prior to 6 min if the patient chooses to stop or the tester terminates testing (indications for terminating exercise as per the ATS guidelines include the following: severe exhaustion or shortness of breath, wheezing, dizziness, chest pain or muscle cramps) [33]. At no point will the patient be encouraged to continue beyond the level at which they wish to stop.

#### Well-being

The Functional Assessment of Chronic Illness Therapy (FACIT) questionnaires have been validated in studies of cancer management and are designed to encompass a range of psychosocial factors [35]. Like the EORTC QLQ-C30, these questionnaires are also a patient reported measure of QoL. However these questionnaires focus on aspects of well-being including physical, social, emotional and functional well-being subscales. Here, the lymphoma-specific scale (FACT-Lym) will be used (comprising the general or FACT-G questionnaire plus a lymphoma subscale. The FACT-Lym has good internal consistency and validity [35]. All questions are answered on a five-point scale and scoring guidelines are recommended together with the questionnaire [36].

#### Psychological status

The Hospital Anxiety and Depression Scale (HADS) is a valid tool for assessing the severity of anxiety disorders and depression in various populations including psychiatric, primary care patients and the general population [37]. The HADS is also recommended in cancer settings [38]. Questions are answered using a four-point scale and higher scores represent higher levels of anxiety and depression.

#### End of study questionnaire

During their final assessment at twelve weeks, participants will be invited to complete the end of study questionnaire. The open questions aim to explore their thoughts and feelings about the intervention programme, and to analyse their views on their particular intervention including preferences, perceived advantages and disadvantages and reasons for participation and adherence.

### Data analysis

Data will be entered into the Microsoft Excel (2013) database by SH and analysed by all authors using the IBM SPSS version 22 (SPSS, Inc., Chicago, IL) statistical software package. The intent-to-treat principle will be applied and the significance level will be set at 0.05. Descriptive statistics will be used to present baseline demographic characteristics. To compare difference in the primary outcome measure, QoL score between the two groups post-intervention, ANCOVA (analysis of covariance) will be used, with pre-test values as covariate to adjust for any baseline differences. Data will be analysed to check fit for ANCOVA assumptions. Post-hoc paired-samples t-tests for pre-post intervention will be tested within groups with Bonferroni corrections. Missing data will be treated as recommended by scoring guidelines of questionnaires [24, 30]. These analyses will be repeated for secondary outcome measures, providing data fit normal distribution and other assumptions for ANCOVA. The influence of potential confounding factors such as age, gender, number of co-morbidities and ECOG status will be explored using correlation analyses. Adherence and drop-out rates will also be analysed. Qualitative data from the end of study questionnaire will be analysed for codes and themes using qualitative content analysis [39].

## Discussion

A large proportion of lymphoma survivors continue to experience unmet needs following treatment [1–3]. Long-term and late effects are often overlooked, and survivors do not routinely receive advice or interventions to target these and maximise well-being [40]. Previous studies have supported the use of both relaxation and exercise interventions to treat physical and psychosocial complaints of cancer survivors [9–12]. No trial to date has compared efficacy of these two interventions in lymphoma survivors.

The REIL study aims to study a sample of lymphoma survivors shortly post-chemotherapy, and compare the effectiveness of relaxation and exercise on improving QoL. This study was developed to address some issues highlighted in survivorship literature including focus on survivors of cancers other than breast or prostate, emphasis on the transition phase immediately following treatment, addressing physical, psychological and social needs of lymphoma survivors, use of patient-reported outcome measures, self-management, and moving from control groups to comparison between two interventions.

In this study lymphoma patients in remission will be recruited within six weeks of their last chemotherapy session. Patients during this early phase of survivorship who have completed initial cancer treatment have been relatively neglected [19], and this period immediately following treatment has been identified as a key time to address side effects of treatment and facilitate return to pre-morbid health [41]. Studies on cancer survivors have indicated that they would prefer to begin an exercise programme immediately or soon after treatment [42], and at this point the focus can move from the disease and treatment to ‘moving on’ to the next phase of life [43].

While the additional psychosocial benefits of supervised group classes for cancer survivors have been recognised [44], the need for a shift towards self-management in cancer survivorship has been highlighted [6, 7]. Here participants are supported to self-manage in a variety of ways, but the onus will be on them to initiate contact with the healthcare professionals for additional support when needed [7, 8]. Both the relaxation and exercise programmes in this study are designed to be performed independently and participants will be supplied with resources to allow convenient home-based performance, including written instructions and advice. Participants will be able to contact the investigator whenever required. This study will provide results including adherence and drop-out rates that are more generalizable to ‘real-world’ lymphoma survivors than controlled clinic-based research. Home-exercise programmes also afford patients better scheduling flexibility, familiar surroundings, family support and reduced travel requirements [45].

It will not be possible to stratify participants by factors such as age, gender, etc. during randomization due to the small sample size. However, sample size was calculated using reference values (including standard deviation) and power calculations recommended by the EORTC reference manual [23]; similar trials on the effects of exercise in cancer patients have been carried out with comparable sample sizes, and these were able to detect significant results [9].

In addition to quantitative data from outcome measures, the end of study questionnaire will explore participants’ perceptions and feelings towards an intervention programme post-chemotherapy. Such information will highlight behaviours and patterns in this sample of lymphoma survivors including reasons for participation, adherence and non-adherence, and any preferences; it is anticipated that this data will ultimately help in the promotion of a healthy lifestyle in lymphoma and cancer survivors.

The authors will aim to publish results from this study to add to the evidence to inform healthcare professionals on effective interventions to improve QoL of lymphoma survivors.

## Conclusion

It is well documented that lymphoma survivors commonly suffer from consequences of treatment such as pain, fatigue, decreased function, anxiety and depression which have a negative impact on their quality of life. However, there is no standardize care pathway recommended for lymphoma survivors following chemotherapy.

The current proposed REIL study aims to determine the more effective intervention of two in improving QoL in lymphoma survivors. It is anticipated that results from this preliminary study will help build towards the development of feasible and effective practice guidelines to improve QoL of lymphoma survivors post-chemotherapy.

## Data Availability

The written material and datasets used and/or analysed during the current study will be available from the corresponding author on reasonable request.

## Abbreviations

6MWT: Six Minute Walk Test
ACSM: American College of Sports Medicine
ANOVA: Analysis of Variance
ATS: American Thoracic Society
BMI: Body Mass Index
BP: Blood Pressure
CD: Compact Disc
COPD: Chronic Obstructive Pulmonary Disease
ECOG: Eastern Cooperative Oncology Group
EORTC QLQ-30: European Organisation for Research and Treatment of Cancer – QoL core questionnaire
FACIT: Functional Assessment of Chronic Illness Therapy
FACT-Lym: Functional Assessment of Cancer Therapy - Lymphoma
FER: Forced Expiratory Rate
FEV1: Forced Expiratory Volume
FVC: Forced Vital Capacity
HADS: Hospital Anxiety and Depression Scale
HOOP: Haematology and Oncology Out-Patients
HR: Heart Rate
NCSI: National Cancer Survivorship Initiative
NHS: National Health Service
PEF: Peak Expiratory Flow
QoL: Quality of Life
REIL: Relaxation and Exercise In Lymphoma
RPE: Rate of Perceived Exercise
TIDieR: Template for Intervention Description and Replication
UK: United Kingdom
